# Mitochondrial dysfunction and autophagy activation underlie NK cell impairment induced by Cannabis

**DOI:** 10.1371/journal.pone.0350750

**Published:** 2026-06-24

**Authors:** Andrée-Ann Bolduc, Tony Tremblay, Mikhlid H. Almutairi, Abdelhabib Semlali, Lionel Loubaki

**Affiliations:** 1 Héma-Québec, Medical Affairs and Innovation, Québec, Québec, Canada; 2 Department of Biochemistry, Microbiology and Bioinformatics, Laval University, Québec, Québec, Canada; 3 Department of Zoology, College of Science, King Saud University, Riyadh, Saudi Arabia; 4 Groupe de Recherche en Écologie Buccale, Faculté de Médecine Dentaire, Université Laval, Québec, Québec, Canada; Nuclear Science and Technology Research Institute, IRAN, ISLAMIC REPUBLIC OF

## Abstract

Cannabis use continues to rise in Canada, prompting concerns due to its potential impact on immune function. This study investigated the effect of a cannabis joint extract (CJE) on natural killer (NK) cells and explored the mechanisms underlying its potential anti-inflammatory properties. Peripheral blood mononuclear cells (PBMCs) were exposed to varying concentrations of CJE to assess cytotoxicity. Flow cytometry was employed to evaluate oxidative stress, autophagy, mitochondrial membrane potential, caspase-3 activation, and DNA damage. Additionally, NK cell cytotoxicity, migration, and adhesion were analyzed. Data indicated that CJE exposure led to dose-dependent cytotoxicity in NK cells, primarily through apoptosis. Specifically, at a concentration of 3 μg/mL, CJE significantly increased reactive oxygen species (ROS), autophagy markers, caspase activation, and DNA damage, while reducing mitochondrial membrane potential. Moreover, CJE impaired NK cell-mediated killing of HeLa cells, though their migratory and adhesive abilities were unaffected. These findings evidence that cannabis can detrimentally affect NK cell viability and function via mechanisms involving autophagy and caspase-dependent apoptosis.

## Introduction

Cannabis, also known as marijuana, is a complex plant comprising approximately 500 phytochemicals, including at least 60 identified phytocannabinoids. These compounds have demonstrated a range of therapeutic effects, acting as analgesics, anti-inflammatory agents, anti-emetics, and anticonvulsants, while also influencing muscle tone, mood, cognition, and appetite [1,2].

Cannabinoids exert their biological effects primarily through the endocannabinoid system (ECS), which includes cannabinoid receptors type 1 (CB1) and type 2 (CB2), their endogenous ligands (endocannabinoids), and the enzymes responsible for their synthesis and degradation [3]. Beyond the ECS, cannabinoids also interact with various non-cannabinoid receptors and ion channels, modulating receptor-independent pathways. For example, they can delay the reuptake of endocannabinoids and neurotransmitters such as anandamide and adenosine and influence the binding of certain G-protein-coupled receptors to their ligands [3].

In recent years, increasing attention has been paid to the immunomodulatory properties of cannabinoids, although their effects on immune responses remain complex and sometimes seemingly contradictory. On one hand, several cannabinoids—particularly CBD, CBG, or specific cannabinoid combinations—have been shown to exert anti-inflammatory and immunosuppressive effects by reducing leukocyte migration, decreasing the production of inflammatory mediators such as nitric oxide and cytokines, and limiting immune cell activation [1,4–8]. On the other hand, accumulating evidence indicates that cannabinoids may also promote pro-inflammatory responses under certain conditions. These effects appear to depend on multiple factors, including the specific cannabinoid, its concentration, the target cell type, receptor engagement, and its metabolic conversion into bioactive lipid mediators. In particular, some studies have reported increased oxidative stress, enhanced secretion of pro-inflammatory cytokines, and stimulation of eicosanoid production, including prostaglandins and leukotrienes, following cannabinoid exposure [9–13]. Altogether, these findings support the concept that cannabinoids act as context-dependent immunomodulators rather than uniformly anti-inflammatory or pro-inflammatory agents.

This study investigates the effects of a cannabis joint extract (CJE) on NK cell function, a critical component of the innate immune system. NK cells play a vital role in identifying and eliminating infected or abnormal cells, and any disruption in their survival or function could have significant consequences for immune competence [14]. The literature on cannabis and NK cells presents conflicting results. NK cells express both CB1 and CB2 receptors, and CB2-deficient mice have been shown to exhibit increased pulmonary NK cell populations [11,15]. An observational study involving high school and university students who regularly consumed cannabis reported a marked reduction in the functional diversity of peripheral blood mononuclear cell subsets, including T and B lymphocytes, with NK cells being most affected [16]. Additionally, administration of delta-9-tetrahydrocannabinol (THC)—the primary psychoactive cannabinoid in cannabis—led to reduced splenic NK activity in mice without affecting cell viability or target cell binding [17]. THC has also been shown to inhibit NK activity in both mice and humans without impairing proliferation [18,19]. In contrast, cannabidiol (CBD), the major non-psychoactive cannabinoid, has been reported to enhance NK cell activity and promote anti-tumor responses [20].

These divergent findings underscore the need for further investigation into the mechanisms by which cannabis compounds influence NK cell survival and function. Building on our previous work, which demonstrated that a cannabinoid mixture affects B cell fate and function, we sought to validate these observations using a real cannabis joint extract (CJE) and NK cells, given their crucial role in immune defense.

## Materials and methods

### Isolation and storage of peripheral blood mononuclear cells

This study received approval from Héma-Québec’s Research Ethics Committee (CER#2020−010), and written informed consent was obtained from all participants. Recruitment of participants for this study began on April 5, 2023, and ended on June 25, 2025. No minor participants were included in this study.

Whole blood (450 mL) was collected using the Leukotrap WB system (Haemonetics, Braintree, MA, USA), following the manufacturer’s protocol. Immediately after donation, peripheral blood mononuclear cells (PBMCs) were isolated by density gradient centrifugation using Ficoll-Paque (Cytiva, Vancouver, BC, Canada) and Leucosep tubes (Greiner Bio-One, Monroe, NC, USA), following the manufacturer’s instructions. The PBMCs were then washed with DPBS (Thermo Fisher Scientific, Waltham, MA, USA) supplemented with 0.25% human albumin (CLS Behring, Ottawa, ON, Canada). Subsequently, the cells were resuspended in a plasmalyte solution (Baxter, Mississauga, ON, Canada) containing 5% human albumin and 18% CryoSure-Dex40 (WAK-Chemi Medical, Steinbach, Germany), aliquoted, and cryopreserved in liquid nitrogen for future use.

### Preparation of the cannabis joint extract

Cannabis joints of the Orchid variety (containing 10.4% THC and 14.7% CBD per 500 mg) were obtained from the Société Québécoise du Cannabis (SQDC), the sole authorized distributor in the province of Québec. The cannabis joint extract (CJE) was prepared using a passive extraction method [21]. Briefly, the cannabis material was removed from the joint, weighed, and ground into a fine powder in a mortar under liquid nitrogen. The resulting powder was weighed again and resuspended in 3–5 mL of 100% DMSO, followed by a 18h incubation at 4 °C. DMSO was selected based on cannabinoids high solubility in this solvent and less cytotoxicity at low concentration [22,23]. The mixture was then centrifuged, and the supernatant was collected and filtered 0.22 µm to make it sterile. The final cannabis filtrate was stored at −20 °C until use in natural killer (NK) cell assays.

### Cell culture and PBMC exposure to CJE

PBMCs were thawed using the ThawSTAR™ system (BioLife Solutions, Bothell, WA, USA) and resuspended in RPMI 1640 medium (Thermo Fisher) supplemented with 20% fetal bovine serum (FBS; Thermo Fisher) and 1X penicillin-streptomycin (PEN/STREP; Sigma-Aldrich, St. Louis, MO, USA). The cell suspension was centrifuged for 10 minutes at 600 × g, after which the supernatant was discarded. Cells were then resuspended at a concentration of 1x10⁶ cells/mL in RPMI medium containing 20% FBS and 1X PEN/STREP. The cell suspension was seeded into 12-well plates (Sigma-Aldrich) and incubated for 3 hours at 37 °C with 5% CO₂. This interval was selected based on validated commercial leucocytes adhesion assays, which typically rely on short adhesion periods (2–4 h). Furthermore, published studies have demonstrated that PBMCs maintain stable viability and recovery for up to 8 hours post‑thaw, whereas extended incubation (e.g., 16 h) significantly decreases viability [24]. Short recovery periods (1–3 h) after thawing are also recommended in standardized thawing SOPs to minimize metabolic drift and preserve cellular functionality. Therefore, the 3‑hour recovery period used in this study falls within experimentally validated windows and ensures methodological robustness while minimizing pre‑activation and variability

Following this incubation, various concentrations of CJE, ranging from 1.5 to 24 µg/mL, were added to the appropriate experimental conditions. As a vehicle control, an equivalent volume of dimethyl sulfoxide (DMSO; Millipore Sigma, Oakville, ON, Canada) corresponding to the highest cannabis concentration was used, given that the extract was prepared in DMSO. Cells were then incubated 18h at 37 °C with 5% CO₂. This incubation period was selected based on the previous cannabis-related blood donation deferral policy in place at our institution.

### Cytotoxicity assay

Following cell culture and exposure to the CJE, PBMC cytotoxicity was evaluated by measuring lactate dehydrogenase (LDH) release in the culture supernatant, as previously described by Semlali et al.  [25]. Briefly, PBMCs were re-suspended in RPMI medium supplemented with 5% FBS and 1X PEN/STREP at a concentration of 1x10⁵ cells/mL and seeded into 12-well plates (Sigma-Aldrich). Cells were incubated for 3 hours at 37 °C with 5% CO₂ prior to the addition of CJE or DMSO, followed by 18h incubation under the same conditions. Supernatants were then collected for LDH quantification using the CyQUANT LDH Cytotoxicity Assay Kit (Cat#C20300, Thermo Fisher), in accordance with the manufacturer’s instructions.

### Flow cytometry analysis of cell viability

In addition to LDH quantification, the viability of natural killer (NK) cells (CD3 ⁻ CD56⁺) was assessed by evaluating the expression of 7-Aminoactinomycin D (7-AAD; Thermo Fisher). Following exposure of PBMCs to the CJE, cells were harvested and distributed into flow cytometry tubes (500,000 cells per tube), then resuspended in Dulbecco’s phosphate-buffered saline (DPBS; Thermo Fish-er) supplemented with 2% FBS. After centrifugation for 5 minutes at 500 × g, the supernatant was discarded, and the cell pellet was gently vortexed. Cells were then stained with anti-CD3-APC (Clone SK7; Thermo Fisher) and anti-CD56-PE (Clone MEM-188; Thermo Fisher), followed by incubation in the dark for 30 minutes at room temperature. After staining, cells were washed with DPBS + 2% FBS, centrifuged again for 5 minutes at 500 × g, and resuspended. The viability marker 7-AAD was then added, and cells were incubated for 10 minutes at 4 °C. Finally, samples were resuspended in 350 μL of DPBS + 2% FBS and analyzed using a BD Accuri C6 flow cytometer (BD Biosciences). Data analysis was performed using FCS Express 6 software (De Novo Soft-ware, Los Angeles, CA, USA).

### Assessment of the oxidative stress response

To evaluate oxidative stress responses in NK cells, PBMCs—either exposed or not to 3 µg/mL of CJE—were stained with anti-CD56-APC-eFluor (Clone CMSSB; Thermo Fisher) and anti-CD3-APC (Clone SK7; Thermo Fisher), then treated with CellROX™ Oxidative Stress Reagent (Cat# C10492, Thermo Fisher) following the manufacturer’s protocol. Flow cytometry data were acquired using the Attune™ flow cytometer (Thermo Fisher) and analyzed with FCS Express 6 software (De Novo Software). The gating strategy involved identifying all cell populations, with NK cells defined as CD3 ⁻ /CD56 ⁺ , and oxidative stress-positive cells identified as ROS⁺ within the PBMC population.

### Mitochondrial membrane potential assay

To investigate changes in mitochondrial membrane potential in NK cells, PBMCs—either untreated or exposed to 3 µg/mL of CJE—were stained using the MitoProbe DiOC₂(3) Assay Kit (Cat# M34150, Thermo Fisher), in combination with anti-CD56-APC-eFluor (Clone CMSSB; Thermo Fisher) and anti-CD3-APC (Clone SK7; Thermo Fisher). Flow cytometry data were acquired using the Attune flow cytometer (Thermo Fisher) and analyzed with FCS Express 6 software (De Novo Software). The gating strategy involved identifying all cell populations, with NK cells defined as CD3 ⁻ /CD56⁺ and cells exhibiting altered mitochondrial membrane potential identified as DiOC₂(3)⁺ within the PBMC population of both control and CJE-treated samples.

### Activated Caspase-3 assay

To detect activated caspase-3 in NK cells, the Cleaved Caspase-3 Staining Kit (FITC; Cat# ab65613, Abcam, Toronto, ON, Canada) was used in combination with anti-CD56-APC-eFluor 680 (Clone CMSSB; Thermo Fisher) and anti-CD3-APC (Clone SK7; Thermo Fisher), as previously described. Flow cytometry data were acquired using the Attune flow cytometer (Thermo Fisher) and analyzed with FCS Express 6 software (De Novo Software). The gating strategy was as follows: all living cells were gated, and CD3-/CD56 + /Caspase-3 + cells were identified in the PBMC population.

### Assessment of DNA damage

Following 18h exposure of PBMCs to CJE, cells were harvested and counted. A total of 5x10⁵ cells were transferred into flow cytometry tubes, resuspended in DPBS supplemented with 2% FBS, and centrifuged for 5 minutes at 500 × g at room temperature. Cells were then fixed with 1.5% paraformaldehyde (Sigma-Aldrich) for 20 minutes at room temperature, followed by a second wash with DPBS + 2% FBS. After fixation, cells were permeabilized by incubation in 90% methanol for 20 minutes at 4 °C. Following another wash, cells were stained with 5 µL of Alexa Fluor 488-conjugated γH2AX antibody (Clone N1-431; BD Biosciences), along with anti-CD56-APC-eFluor (Clone CMSSB; Thermo Fisher) and anti-CD3-APC (Clone SK7; Thermo Fisher). A final wash with DPBS + 2% FBS was performed prior to data acquisition using the Attune NxT flow cytometer (Thermo Fisher). The gating strategy was as follows: first, all CD3-CD56 + cells were selected using a dot plot, and then γH2AX+ cells were identified within this population.

### Assessment of autophagy

To evaluate autophagy in viable NK cells, PBMCs—either untreated or exposed to 3 µg/mL of CJE—were stained using the Autophagy Assay – Red Fluorescent (Cat# SKU9156, ImmunoChemistry Technologies, Davis, CA, USA), along with anti-CD56-APC-eFluor (Clone CMSSB; Thermo Fisher) and anti-CD3-APC (Clone SK7; Thermo Fisher). In parallel, NK cells isolated from PBMCs previously treated or not with CJE were purified using the EasySep Human NK Cell Enrichment Kit (Cat# 19055; STEMCELL Technologies Inc., Vancouver, BC, Canada). To assess the purity of the isolated NK cell population, cells were stained with anti-CD3-APC (Clone SK7), anti-CD56-PE (Clone MEM-188), and the viability marker 7-AAD (all from Thermo Fisher), followed by flow cytometry analysis using the BD Accuri C6 flow cytome-ter (BD Biosciences). Data were analyzed using FCS Express 6 software (De Novo Software).

Purified NK cells were subsequently used to investigate the differential expression of autophagy-related genes following exposure to CJE. Real-time PCR was performed using the RT² Profiler PCR Array for Human Autophagy (Cat# 330231; PAHS-084Z; Qiagen, Mississauga, ON, Canada). Additionally, PBMCs were pretreated with 100 µM chloroquine diphosphate for 1 hour prior to CJE exposure, and NK cell viability was assessed thereafter. Flow cytometry data were acquired using the Attune NxT flow cytometer (Thermo Fisher) and analyzed with FCS Express 6 software. The gating strategy involved selecting CD3 ⁻ /CD56 ⁺ cells and identifying Red Autophagy⁺ cells within this population.

### Assessment of the killing capacity of NK cells

NK cells isolated from PBMCs previously exposed or not to CJE were purified using the EasySep Human NK Cell Enrichment Kit (STEMCELL Technologies). The purity of the isolated NK cell population was confirmed by flow cytometry, as described above. Purified NK cells were then co-cultured with HeLa cells to assess their cytolytic activity. Specifically, NK cells (4x10⁵ cells/well) and HeLa cells (2x10⁴ cells/well) were seeded in 96-well plates (Corning, Manassas, VA, USA) at a 20:1 effector-to-target ratio in a final volume of 200 µL of RPMI medium supplemented with 20% FBS. The co-culture was incubated for 72 hours at 37 °C with 5% CO₂.

Following incubation, HeLa cells were detached using TrypLE Express Enzyme (Thermo Fisher), collected, and quantified by flow cytometry using the BD Accuri C6 flow cytometer (BD Biosciences). Briefly, cells were transferred into 3.5 mL flow cytometry tubes and stained with anti-CD56-PE (Clone MEM-188; Thermo Fisher), anti-CD45-APC (Clone HI30; BD Biosciences), and the viability marker 7-AAD (Thermo Fisher). After a 30-minute incubation at room temperature, 300 µL of DPBS + 2% FBS was added to each sample prior to analysis. The percentage of dead HeLa cells was calculated with the following formula:


$$\begin{array}{c} \%\;Dead\;Hela\;cells=\text{1}\text{0}\text{0}-[(\frac{\text{Number of Hela cells recovered after coculture}}{\text{Number of Hela cells seeded in the well}})\;x\;\text{1}\text{0}\text{0}\%] \end{array}$$

### Assessment of Granzyme B and CD107a expression

Following 18h exposure of PBMCs to CJE, cells were harvested and counted. A total of 5x10⁵ cells were transferred into flow cytometry tubes, resuspended in DPBS supplemented with 2% FBS, and centrifuged for 5 minutes at 500 × g at room temperature. Cells were then fixed with 1.5% paraformaldehyde (Sigma-Aldrich) for 20 minutes at room temperature, followed by a second wash with DPBS + 2% FBS. After fixation, cells were permeabilized by incubation in 90% methanol for 20 minutes at 4 °C. Following another wash, cells were stained with 5 µL of either FITC-conjugated anti-Granzyme B antibody (Clone GB11; BD Biosciences) or PE-conjugated anti-CD107a antibody (Clone GB11; BD Biosciences), in combination with anti-CD56-APC-eFluor (Clone CMSSB; Thermo Fisher) and anti-CD3-APC (Clone SK7; Thermo Fisher). A final wash with DPBS + 2% FBS was performed prior to data acquisition using the Attune NxT flow cytometer (Thermo Fisher). The gating strategy involved selecting CD3 ⁻ /CD56 ⁺ cells in a dot plot, followed by identification of Granzyme B⁺ or CD107a⁺ cells within this population.

### Assessment of NK cells’ migration

Chemotactic migration of NK cells toward recombinant human SDF-1 (rhSDF-1) was assessed using Transwell inserts (6.5 mm diameter, 8 µm pore size; Thermo Fisher). Viable NK cells (between 5x10³ and 1 × 10⁴), isolated from PBMCs previously ex-posed or not to CJE, were resuspended in 100 µL of unsupplemented RPMI medium and seeded into the upper chamber of the Transwell. The inserts were placed into wells containing 600 µL of RPMI medium supplemented with 250 ng/mL of rhSDF-1 (Miltenyi Biotec, Waltham, MA, USA). The plates were incubated 18h at 37 °C with 5% CO₂. The following day, Transwell inserts were removed, and the cells that had migrated into the lower chamber were collected and counted using the NC-250 automated cell counter (ChemoMetec). The percentage of NK cell migration was calculated using the following formula:


$$\begin{array}{c} \%\;of\;migration=[\frac{Number\;of\;NK\;cells\;collected}{Number\;of\;NK\;cells\;seeded}]\;x\;\text{1}\text{0}\text{0} \end{array}$$

### Assessment of NK Cells’ Adhesion

Following 18h exposure of PBMCs to CJE, NK cells were purified and seeded into 24-well microplates (Corning) previously coated with human fibronectin (20 µg/mL; VWR, Mississauga, ON, Canada). Between 3 x10⁴ and 5 x 10⁴ viable NK cells were added per well and allowed to adhere for 2 hours at 37°C with 5% CO₂. After incubation, the supernatant was collected, and the number of non-adherent cells was quantified using the NC-250 automated cell counter (ChemoMetec, Bohemia, NY, USA). The percentage of NK cell adhesion was calculated using the following formula:


$$\begin{array}{c} \%\;of\;adhesion=\text{1}\text{0}\text{0}-[(\frac{Number\;of\;NK\;cells\;collected}{Number\;of\;NK\;cells\;seeded})\;x\;\text{1}\text{0}\text{0}] \end{array}$$

### Assessment of NK cells signaling pathways

Signaling pathways involved in NK cell development and function were assessed by flow cytometry, following the same protocol used for DNA damage analysis (as previously described), with the exception of the antibody panel. For this assay, 1x10⁴ cells were stained with Alexa Fluor 647-conjugated antibodies targeting phosphorylated signaling proteins, including phospho-NF-κB (Ser529; Clone B33B4WP), phospho-ERK1/2 (Clone 20A), phospho-p38 (Clone pT180/pY182), phospho-STAT5 (Clone pY694), and phospho-AKT1 (Ser473; Clone SDRNR), as well as PE-conjugated phospho-mTOR (Ser2448; Clone MRRBY), all obtained from BD Biosciences. These antibodies were used in combination with anti-CD3-APC (Clone SK7) and anti-CD56-APC-eFluor (Clone CMSSB; Thermo Fisher). Flow cytometry data were acquired using the BD Accuri C6 flow cytometer (BD Biosciences) and analyzed with FCS Express 6 software (De Novo Software). The gating strategy involved identifying CD3 ⁻ /CD56 ⁺ NK cells and quantifying the proportion of cells positive for each phosphorylated signaling marker within this population**.**

### Statistical analysis

All analyses were performed using GraphPad Prism 10.2.3 (GraphPad, San Diego, CA, USA). Values are presented as mean ± standard error of the mean (SEM), and statistical analyses were carried out using a Kruskal-Wallis test (nonparametric ANOVA), Wilcoxon matched-pairs signed rank tests, and a Mann-Whitney test when appropriate. A p-value below 0.05 was considered statistically significant.

## Results

### Exposure to a cannabis joint extract affects NK Cell viability

**Fig 1:**
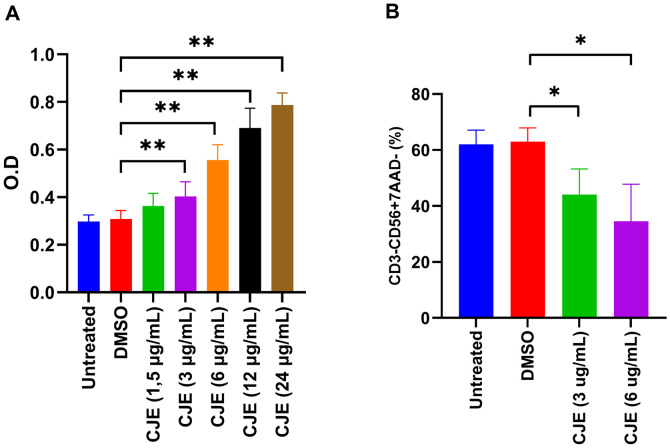
Cannabis joint extract (CJE) reduces PBMC and NK cell viability. **(A)** LDH release in PBMCs exposed to increasing concentrations of CJE, indicating a concentration-associated cytotoxicity. **(B)** Quantification of viable NK cells (CD3 ⁻ CD56 ⁺ 7-AAD⁻) following CJE exposure. Data represent mean ± standard error of the mean (SEM). *p <0.05; n = 4-7 independent experiments; DMSO = dimethyl sulfoxide; CJE = Cannabis joint Extract; OD = optical density (490 nm).

We recently reported that exposure of PBMCs to a cannabinoid mixture induces apoptosis within the leukocyte population, particularly affecting B cells  [26]. To determine whether a whole cannabis joint extract (CJE) exerts similar effects on NK cells, PBMCs were treated with increasing concentrations of CJE, corresponding to estimated bioavailability levels of 2% (1.5 µg/mL), 6% (3 µg/mL), 12.5% (6 µg/mL), 25% (12 µg/mL), and 50% (24 µg/mL)—equivalent to the systemic exposure following inhalation of 1 g of cannabis containing 24% cannabinoids. A concentration-associated increase in lactate dehydrogenase (LDH) release was observed, indicating significant cytotoxicity induced by CJE in PBMCs (Fig 1A). Furthermore, NK cell viability, assessed by 7-AAD exclusion in CD3 ⁻ CD56 ⁺ cells, decreased significantly with increasing CJE concentrations (Fig 1B).

### Exposure to a cannabis joint extract significantly modulates oxidative stress and mitochondrial membrane potential in NK cells

**Fig 2:**
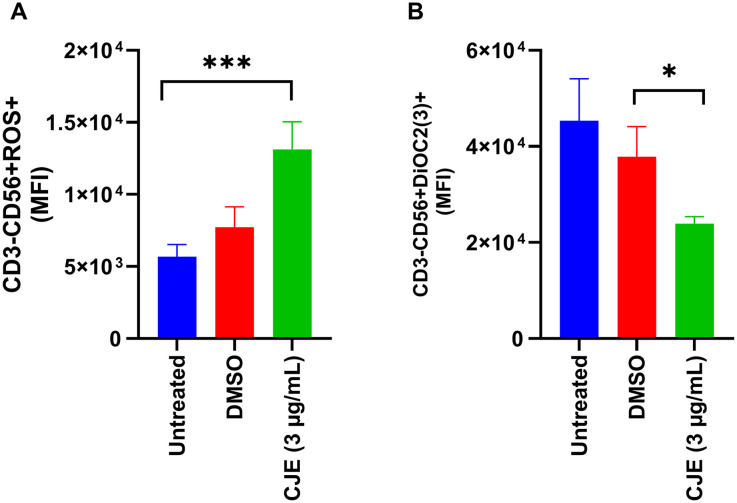
CJE induces oxidative stress and mitochondrial dysfunction in NK cells. PBMCs were exposed or not to 3 µg/mL of CJE, then stained with APC-conjugated anti-CD3 and APC-eFluor680-conjugated anti-CD56 antibodies. **(A)** Intracellular reactive oxygen species (ROS) levels were measured using CellROX™ Oxidative Stress Reagents. **(B)** Mitochondrial membrane potential was assessed using the MitoProbe™ Di-OC₂(3) Assay Kit. All analyses were performed by flow cytometry. Data are presented as mean ± SEM. *p < 0.05; ***p < 0.001; n = 4 independent experiments. DMSO = dimethyl sulfoxide; MFI = median fluorescence intensity.

Cannabinoids has previously been shown to promote apoptosis in immune cells by generating reactive oxygen species (ROS) [11,26]. To investigate whether ROS contribute to CJE-induced NK cell death, PBMCs were exposed to 3 µg/mL of CJE—a concentration at which both viable and non-viable cells were consistently observed. This treatment led to a significant increase in intracellular ROS levels in CD3 ⁻ CD56 ⁺ NK cells, with a nearly twofold elevation compared to untreated controls (Fig 2A). Given the established link between elevated ROS and mitochondrial damage  [27], we next assessed mitochondrial membrane potential in NK cells. Exposure to CJE resulted in a marked reduction—approximately twofold—in mitochondrial membrane potential in CD3 ⁻ CD56 ⁺ cells (Fig 2B).

### Exposure to cannabis joint extract induces apoptosis and DNA damage in NK cells

**Fig 3:**
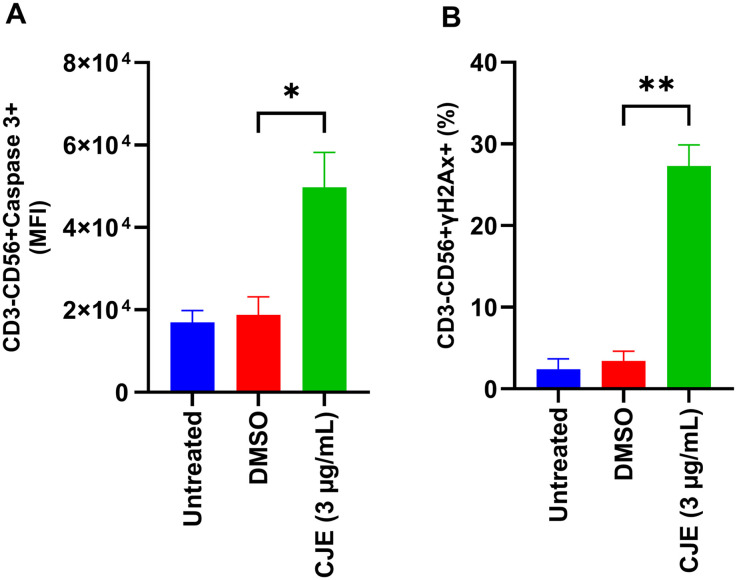
CJE induces apoptosis and DNA damage in NK cells. PBMCs were exposed or not to 3 µg/mL of CJE. **(A)** Activated caspase-3 levels were measured in CD3 ⁻ CD56 ⁺ NK cells using the Caspase-3 Detection Kit and flow cytometry. **(B)** DNA damage was assessed by quantifying γH2AX expression in NK cells via flow cytometry. Data are presented as mean ± SEM. *p < 0.05; **p < 0.01; n = 4-5 independent experiments. Untreated = no exposure; DMSO = dimethyl sulfoxide; CJE = cannabis joint extract; MFI = median fluorescence intensity.

To further elucidate the mechanisms underlying CJE-induced NK cell death, we investigated the involvement of apoptotic pathways and DNA damage. Previous studies, including our own, have highlighted the role of caspase activation in cannabinoid-induced apoptosis [11,26,28]. Flow cytometry analysis revealed a significant increase—approximately threefold—in activated caspase-3 levels in NK cells exposed to CJE (49,755.51 ± 8,447.16 median fluorescence intensity [MFI]) compared to untreated (16,963.4 ± 2,894.87 MFI) and vehicle-treated cells (18,776.98 ± 4,405.86 MFI; Fig 3A). In parallel, CJE exposure markedly elevated the expression of the DNA damage marker γH2AX in NK cells, with an approximately elevenfold increase in the proportion of γH2AX⁺ cells (Untreated: 2.42 ± 1.26%; DMSO: 3.42 ± 1.19%; CJE: 27.27 ± 2.61%; Fig 3B).

### Cannabis joint extract promotes autophagy in NK cells and modulates apoptosis

**Fig 4:**
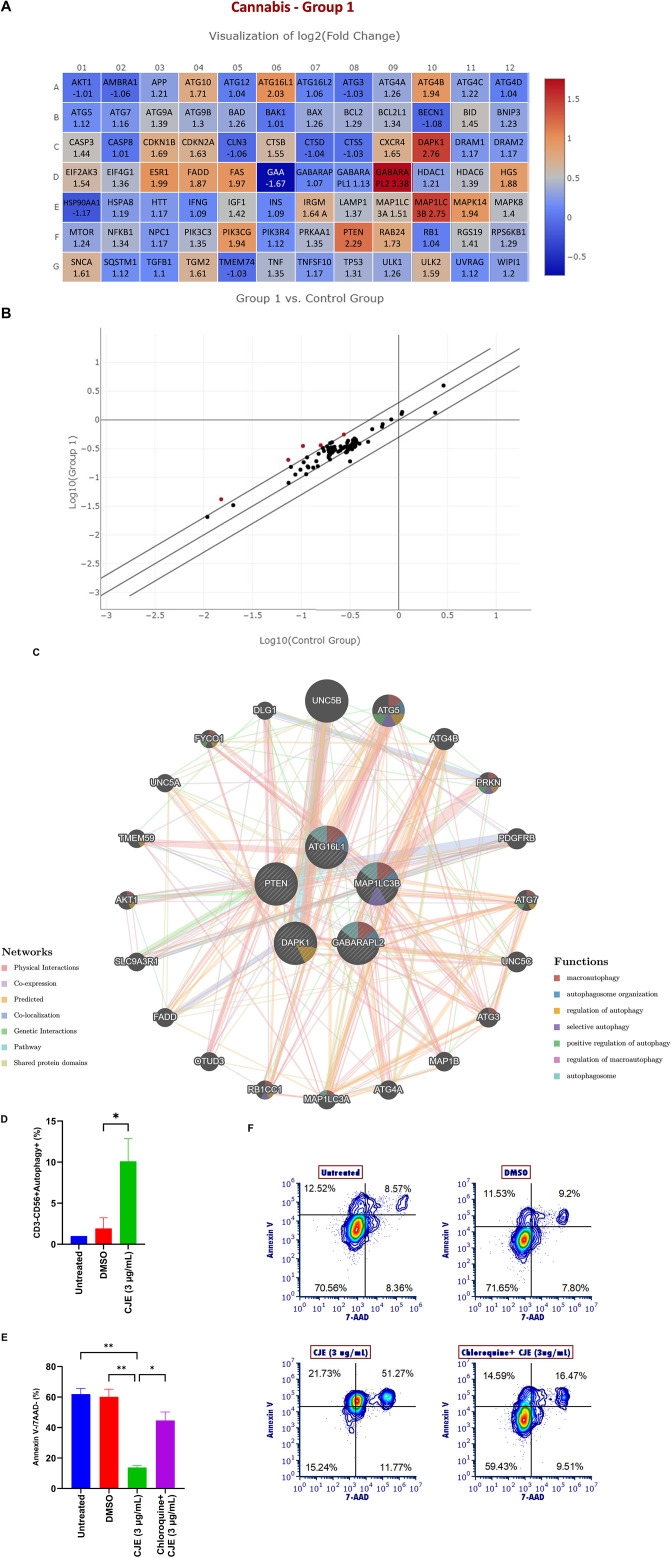
CJE induces autophagy and modulates the viability of NK cells. RNA was extracted from purified NK cells, converted to cDNA, and analyzed by real-time PCR to assess autophagy-related gene expression. **(A–C)** Heatmap and representative gene interaction networks comparing CJE-treated and DMSO control conditions. **(D)** Autophagy levels in viable NK cells were assessed by flow cytometry using the Red Fluorescent Autophagy Assay, in combination with anti-CD3-APC and anti-CD56-APC-eFluor680 antibodies. **(E–F)** PBMCs were pretreated or not with 100 µM chloroquine diphosphate (autophagy inhibitor), followed by exposure to CJE. Apoptosis was evaluated by Annexin V and propidium iodide (PI) staining in CD3 ⁻ CD56 ⁺ NK cells. Data are presented as mean ± SEM. *p < 0.05; **p < 0.01; n = 4 independent experiments. DMSO = dimethyl sulfoxide; CJE = cannabis joint extract.

Autophagy, a cellular process involved in stress adaptation and survival, was investigated in NK cells following exposure to CJE. As shown in Fig 4A–4C, CJE significantly upregulated the expression of autophagy-related genes. Flow cytometry analysis further confirmed a marked increase in autophagic activity in CD3 ⁻ CD56 ⁺ NK cells upon CJE treatment (Fig 4D), suggesting that autophagy may be part of the cellular response to CJE-induced stress. Notably, pretreatment of PBMCs with 100 µM chloroquine diphosphate, an autophagy inhibitor, resulted in a significant reduction—approximately threefold—in CJE-induced apoptosis (Fig 4E and 4F).

### Cannabis joint extract impairs NK cell cytotoxicity without affecting adhesion or migration

**Fig 5:**
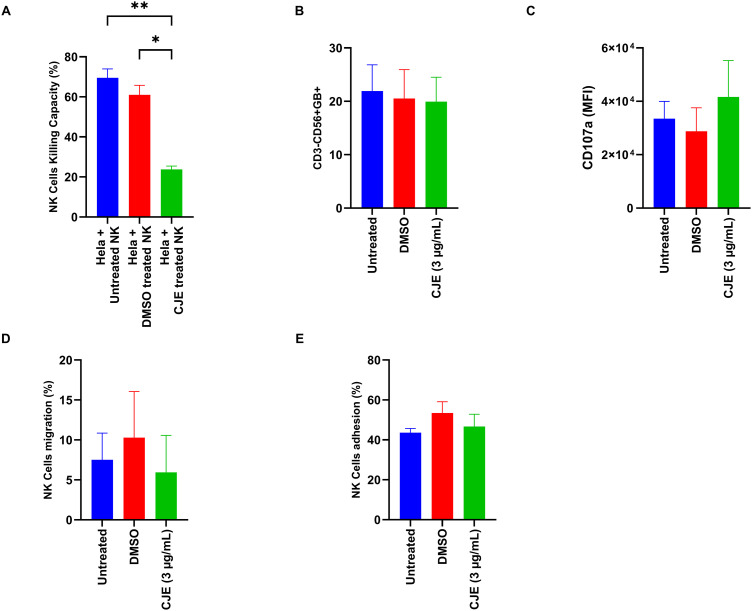
CJE impairs NK cell cytotoxicity without affecting migration or adhesion. PBMCs were either untreated or exposed to 3 µg/mL of CJE, and then NK cells were isolated using the EasySep Human NK Cell Isolation Kit. The isolated NK cells were evaluated for: (A) cytotoxic activity against HeLa cells, **(B)** Granzyme B expression by flow cytometry using anti-Granzyme B staining, (C) migratory response to an SDF-1 gradient, and (D) adhesion capacity on a fibronectin-coated surface. Data are presented as mean ± SEM. *p < 0.05; **p < 0.01; n = 4 independent experiments. DMSO: dimethyl sulfoxide.

The primary role of NK cells is to eliminate abnormal or infected cells that downregulate major histocompatibility complex class I molecules or overexpress ligands for NK cell-activating receptors [29,30]. To evaluate the impact of CJE on NK cell cytotoxic function, purified NK cells were co-cultured with HeLa cells. A significant reduction—approximately threefold—in NK cell-mediated cytotoxicity was observed following CJE exposure (Fig 5A). This cytolytic activity typically involves effector molecules such as Granzyme B and CD107a [31]; however, their expression levels were not significantly altered by CJE treatment (Fig 5C).

In addition to cytotoxicity, NK cell adhesion and migration are crucial for effective immune surveillance and the elimination of target cells  [32,33]. Our results showed that CJE exposure did not significantly affect NK cell migration in response to an SDF-1 gradient (Untreated: 7.67 ± 3.25%; DMSO: 10.29 ± 5.76%; CJE: 5.9 ± 4.63%; Fig 5C). Similarly, no substantial changes were observed in NK cell adhesion to fibronectin-coated surfaces following CJE treatment (Untreated: 45.8 ± 6.35%; DMSO: 57.8 ± 2.25%; CJE: 48.8 ± 9.8%; Fig 5D).

### Cannabis joint extract modulates key signaling pathways in NK cells

**Fig 6:**
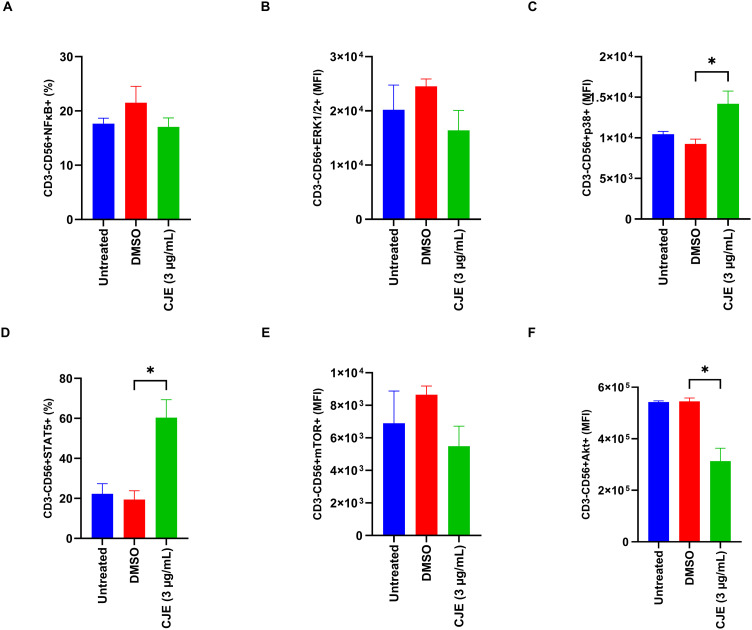
CJE modulates intracellular signaling pathways in NK cells. PBMCs were either untreated or exposed to 3 µg/mL of CJE, then stained with FITC-conjugated anti-CD3 and PE-conjugated anti-CD56 antibodies, along with AlexaFluor-647-conjugated antibodies targeting phosphorylated forms of **(A)** NF-κB, **(B)** ERK1/2, (C) p38, **(D)** STAT5, (E) mTOR, and **(F)** AKT1. Phosphorylation levels were assessed by flow cytometry. Data are presented as mean ± SEM. *p < 0.05; n = 4 independent experiments. DMSO: dimethyl sulfoxide; CJE: cannabis joint extract.

To investigate the intracellular signaling mechanisms affected by CJE, we analyzed the phosphorylation status of several key signaling proteins in NK cells. As shown in Fig 6, CJE exposure significantly increased the phosphorylation of p38 MAPK (Fig 6C) and STAT5 (Fig 6D), while phosphorylation of AKT1 was markedly reduced. In contrast, the phosphorylation levels of NF-κB (Fig 6A), ERK1/2 (Fig 6B), and mTOR (Fig 6E) remained largely unchanged following CJE treatment. These results indicate that CJE selectively modulates signaling pathways in NK cells, which may contribute to the observed alterations in cell viability and effector functions.

## Discussion

We previously demonstrated that a cannabinoid mixture modulates B cell fate and function through the activation of caspase and MAP kinase signaling pathways  [14]. While most studies investigating the immunomodulatory effects of cannabis have focused on isolated compounds such as THC and CBD, limited research has examined the impact of whole cannabis joint extracts (CJE) on immune cells  [17,34,35]. It is now well established that cannabis contains a wide array of bioactive constituents that may act independently or synergistically to influence leukocyte function  [11]. In the present study, we show that CJE profoundly alters NK cell survival and effector functions through multiple mechanisms, with potential implications for immune regulation and cancer immunosurveillance.

Cannabinoids inhaled via combustion are rapidly degraded by pyrolysis, yet they reach peak plasma concentrations within minutes of exposure  [36,37]. The bioavailability of inhaled cannabinoids varies widely, ranging from 2% to 56%  [38–40]. For instance, six minutes after inhaling 13 mg of THC (approximately 2.5 x 10¹⁹ molecules), only 2.8% (1.4 x 10¹⁴ molecules/mL) was detectable in plasma  [41]. Similarly, one minute after intravenous administration of 5 mg of THC (9.55 x 10¹⁸ molecules), plasma concentrations reached 4.28 x 10¹⁴ molecules/mL, meaning only 22% [41]. These findings highlight the rapid clearance of cannabinoids and the substantial gap between administered and circulating doses.

Based on these pharmacokinetic considerations, we exposed PBMCs to a range of CJE concentrations reflecting estimated bioavailability levels. Consistent with our previous findings using a cannabinoid mixture, CJE exposure induced a concentration-associated increase in PBMC cytotoxicity, with a notable reduction in NK cell viability. This observation aligns with the study by El-Gohary et al.  [16], which reported lower counts of PBMCs, T cells, B cells, and NK cells in cannabis users compared to non-users. However, our results diverge from other studies where THC exposure alone did not affect NK cell viability  [34,42]. This discrepancy may be attributed to the use of a whole extract containing both THC and CBD, as suggested by Klein et al.  [34]. Notably, CBD has been reported to enhance NK cell activity and promote anti-tumor responses  [20], underscoring the complexity of cannabinoid interactions.

To further investigate the mechanisms underlying CJE-induced NK cell death, we selected a concentration of 3 µg/mL, which allowed for the simultaneous observation of both viable and apoptotic cells. Given the established role of ROS in cannabinoid-induced apoptosis [11,26,43], we assessed oxidative stress and mitochondrial integrity. CJE exposure significantly reduced mitochondrial membrane potential, leading to increased intracellular ROS levels. These findings are consistent with previous reports of oxidative stress in synthetic cannabinoid users and in B cells [26,44].

The activation of caspase-3 and the upregulation of γH2AX, a marker of DNA damage  [45,46], further support the pro-apoptotic and genotoxic effects of CJE on NK cells. These results suggest that CJE triggers both intrinsic and extrinsic apoptotic pathways, potentially mediated by oxidative stress and mitochondrial dysfunction. Given the interplay between apoptosis and autophagy, we explored the role of autophagy in CJE-induced cell death [47]. Our data demonstrates that CJE activates autophagy in NK cells, as indicated by increased expression of autophagy-related genes and enhanced autophagic activity. Importantly, pretreatment with chloroquine diphosphate, an autophagy inhibitor, partially attenuated CJE-induced apoptosis, indicating that autophagy contributes to NK cell death while also suggesting the involvement of additional pathways.

CJE exposure also significantly impaired NK cell cytolytic function, consistent with previous studies [17,34,42]. However, the expression of Granzyme B and CD107a—key molecules involved in cytotoxicity—remained unchanged, suggesting that the reduction in cytolytic capacity may be independent of these effectors. NK cell activation relies on a balance between stimulatory and inhibitory receptors, such as NKp30, NKp46, NKG2D, and NKG2A  [32]. Future studies should investigate whether CJE modulates the expression or signaling of these receptors, potentially disrupting NK cell activation thresholds.

Despite the observed impairment in cytotoxicity, CJE did not significantly affect NK cell migration or adhesion, two essential processes for effective immune surveillance [32,33]. These findings are in agreement with previous work by Klein et al., who reported that THC does not interfere with effector-target cell binding  [17].

Ultimately, our analysis of intracellular signaling revealed that CJE modulates key pathways involved in regulating NK cells. Specifically, we observed increased phosphorylation of p38 and STAT5, alongside reduced phosphorylation of AKT1. These pathways are known to influence cell survival, proliferation, and immune function, and their modulation by CJE may underlie the observed changes in NK cell behavior.

Collectively, our findings suggest that cannabis use may negatively impact critical aspects of NK cell function and overall immune competence. This raises concerns about the ability of cannabis users to mount effective responses against infections and malignancies. Notably, cannabis use has been associated with increased susceptibility to fungal infections—potentially due to inhalation of Aspergillus spores—as well as a range of immunologic lung disorders  [48–50]. Moreover, the detrimental effects observed in our study occurred at concentrations as low as 3 µg/mL, consistent with findings by Melén et al. [51], indicating that even occasional cannabis use may transiently compromise NK cell viability and function.

## Conclusions

This study provides compelling evidence that exposure to a cannabis joint extract (CJE) significantly alters NK cell viability and function through multiple interconnected mechanisms. We demonstrate that CJE induces oxidative stress, mitochondrial dysfunction, apoptosis, and DNA damage in NK cells, while also activating autophagy and modulating key intracellular signaling pathways. These effects culminate in a marked reduction in NK cell cytotoxic capacity, although their migratory and adhesive properties remain unaffected. Importantly, these findings were observed at concentrations as low as 3 µg/mL, which are within the range of cannabinoid bioavailability following cannabis inhalation. This raises concerns about the potential immunosuppressive effects of cannabis use, even at low or occasional exposure levels. Given the critical role of NK cells in immune surveillance and tumor control, our results underscore the need for further investigation into the long-term impact of cannabis consumption on innate immunity.

Future studies should aim to dissect the contributions of individual cannabinoids and other bioactive compounds present in whole cannabis extracts, as well as explore their effects in vivo. Understanding the molecular and cellular consequences of cannabis exposure is essential for informing public health policies and guiding clinical recommendations, particularly in the context of increasing cannabis legalization and use.
